# GC–MS profiling and antibacterial activity of *Solanum khasianum* leaf and root extracts

**DOI:** 10.1186/s42269-022-00818-9

**Published:** 2022-05-08

**Authors:** Pavani Chirumamilla, Sunitha Bai Dharavath, Shasthree Taduri

**Affiliations:** Department of Biotechnology, Kakatiya University, Warangal, Telangana 506009 India

**Keywords:** *Solanum khasianum*, GC–MS analysis, Antibacterial activity, Phytochemicals, Biological activity

## Abstract

**Background:**

*Solanum khasianum* is an important medicinal herb of the Solanaceae family. The present study was focused to determine the bioactive compounds in *S. khasianum* leaf and root extract by GC–MS analysis and their antibacterial activity by agar well diffusion method.

**Results:**

Sixteen bioactive compounds were detected in leaf extract and thirty-two compounds in root methanolic extract by GC–MS. The major potent compounds identified in leaf and root extracts were heptadecane 9-hexyl (43.65%) and stigmasterol (23.18%). The root extract showed increased antibacterial activity than leaf extract.

**Conclusion:**

These extracts possessed significant antibacterial activity against the tested bacterial isolates in dose-dependent manner. This study provides the phytoconstituents, antibacterial property and scientific evidence for the traditional claim and use of *S. khasianum.*

## Background

Nature is the richest source of several natural therapeutic compounds. Solanaceae, one of the largest plant families with huge and varied secondary metabolites, used in the management of several ailments. The medicinal value of plants can be correlated to different phytochemicals, as they offer a wide diversity of pharmacological activities. Due to these pharmacological properties, a great attention has been derived toward the medicinal plants.

*Solanum khasianum* is a traditional medicinal plant belonging to Solanaceae family. The plant was known to possess potential alkaloids (solasodine, solasonine, solanine, solamargine and khasianine) that represent an alternative source of medicine (Kaunda and Zhang 2019; Chirumamilla et al. 2021). The berries of *S. khasianum* was reported to possess anticancer (Rosangkima and Jagetia 2015), antibacterial (Pavani and Shasthree 2021), anti-inflammatory (Chirumamilla et al. 2022), antioxidant, anti-diabetic and anti-cholinesterase properties (Gogoi et al. 2021). Besides these, the plant is used traditionally to treat several other diseases like filaria, smallpox, whooping cough, rheumatism, trachoma, bronchitis, snake bites, skin and tooth infections (Chirumamilla et al. 2021).

To the best of our knowledge there is no information on the chromatographic analysis of *S. khasianum* leaf and root extracts. Hence, the current study was focused to determine several bioactive compounds in *S. khasianum* leaf and root extracts by GC–MS analysis. The antibacterial property against gram positive and gram negative bacteria isolates was also revealed by agar well diffusion method.

## Methods

### Collection and preparation of plant material

Fresh leaves and roots of *S. khasianum* were collected during the months of April–May from the department greenhouse (18.0264138, 79.5589066). The plant material was washed thoroughly under running tap water, drained and shade dried at room temperature. These samples were ground to fine powder using homogenizer. The powdered plant material was mixed with methanol (1:10 w/v) and incubated at 22 °C in an orbital shaker at 120 rpm for 48 h. The samples were filtered using Whatman no.1 filter paper, evaporated and the crude methanolic extracts were subjected to GC–MS profiling and antibacterial activity.

### Gas chromatography and mass spectroscopy (GC–MS) analysis

Gas chromatography and mass spectrometry were performed to analyze the qualitative and quantitative identification of organic compounds in the given sample. The potential biological compounds of *S. khasianum* leaf and root extracts were analyzed using GC–MS (Agilent: 7890-Jeol: AccuTOF GCV) system coupled with Elite 1 column. Helium gas was used as a carrier gas at 1 ml/min rate of flow, with an injector volume of 2 µl and 280 °C temperature. The oven temperature was raised from 40 to 280 °C with an isothermal for 5 min. The bioactive compounds were identified based on retention time, MS fragment ions generated and the percentage of these bioactive compounds was evaluated from the total peak area. The phytochemicals have been identified by comparing their MS spectrum patterns to the standard mass spectra available at the National Institute of Standards and Technology (NIST) Mass Spectra Database.

### Antibacterial activity

The leaf and root methanolic extract of *S. khasianum* were tested for their antibacterial activity by agar well diffusion method. Luria Bertani (LB) medium was prepared, poured at 20 ml/petridish and allowed to solidify. 24-h-old bacterial cultures (*Bacillus sphaericus, Escherichia coli, Staphylococcus aureus* and *Pseudomonas aeruginosa*) were spread uniformly onto solidified medium. Different concentrations (20, 40, 60 and 80 µg/ml) of *S. khasianum* leaf and root extracts reconstituted in DMSO (dimethyl sulfoxide 10%) and streptomycin standard (10 µg/ml) were loaded into wells and incubated at 37 °C for 24 h. The antibacterial efficacy of *S. khasianum* extracts were observed by measuring the diameter of inhibition zones emerging around the wells. The results of triplicate mean were taken and data was presented as mean ± SD of the respective triplicate.

## Results

**Fig. 1:**
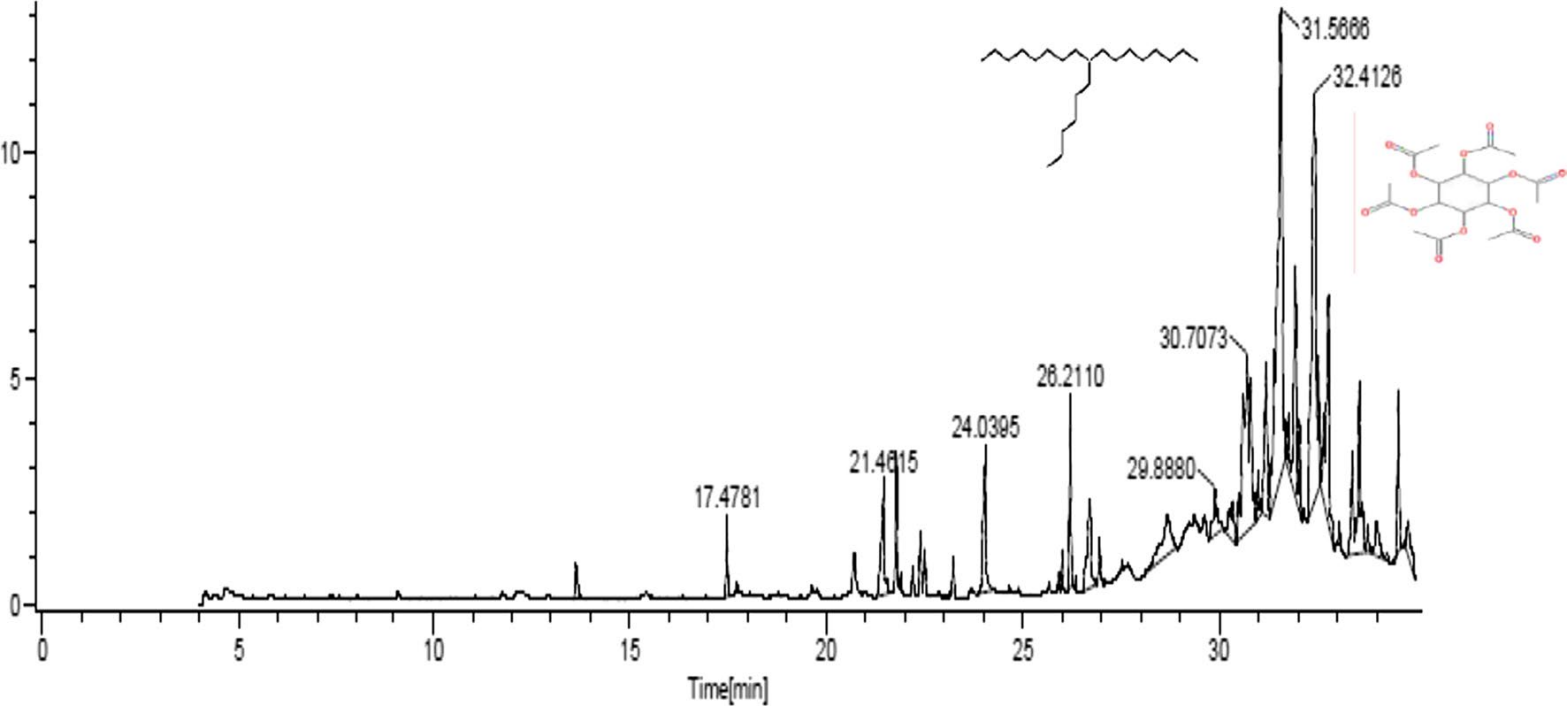
GC–MS chromatogram of leaf methanolic extracts of *Solanum khasianum*. Potent Antifungal agent Heptadecane, 9-hexyl (43.65%) and Myoinositol (15.05%), precursor of several metabolic pathways

**Fig. 2:**
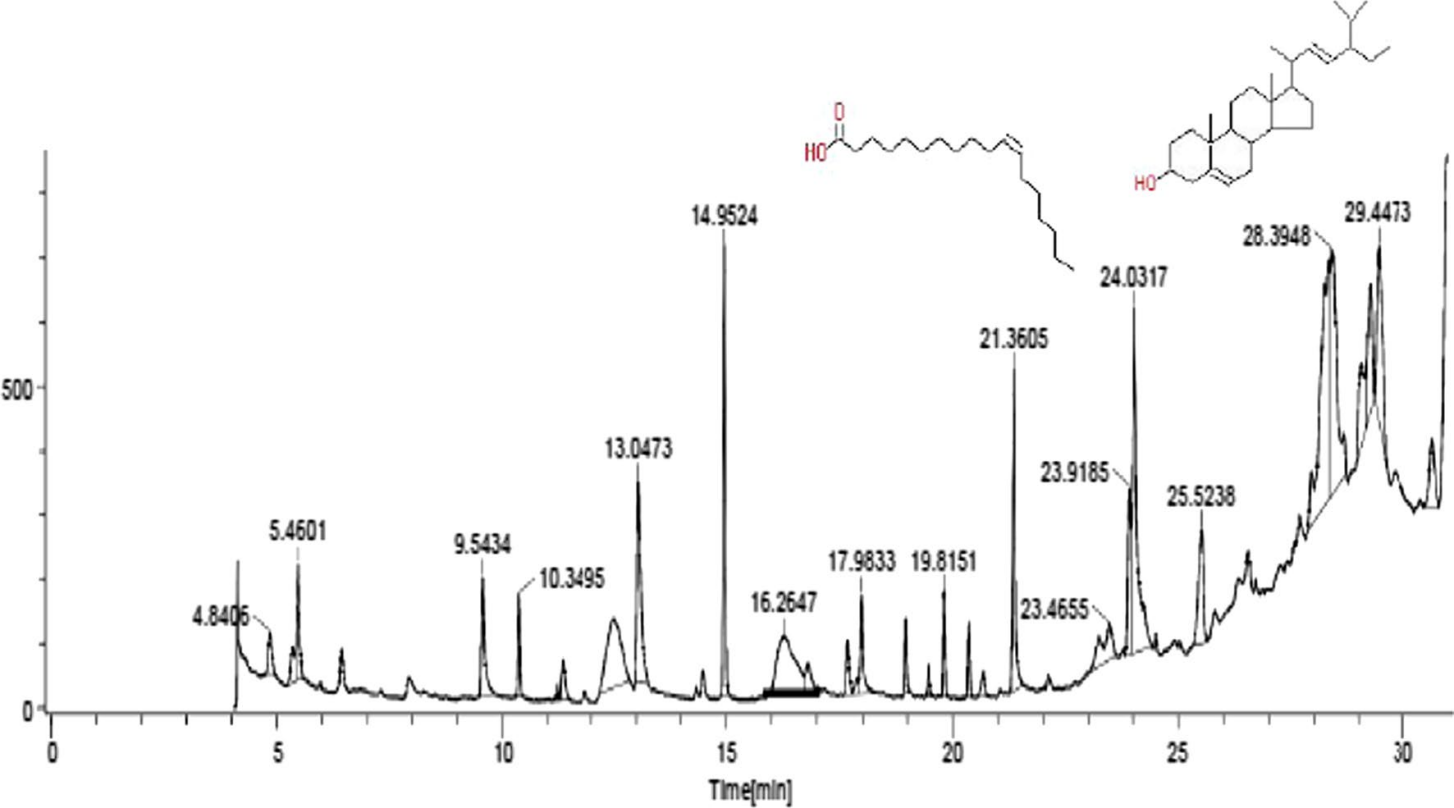
GC–MS chromatogram of root methanolic extracts of *Solanum khasianum*. Stigmasterol (23.18%) with anti-inflammatory, antioxidant, antimicrobial and sedative properties. *cis*-Vaccenic acid (9.07%), potent anti-cancerogenic effect

**Fig. 3:**
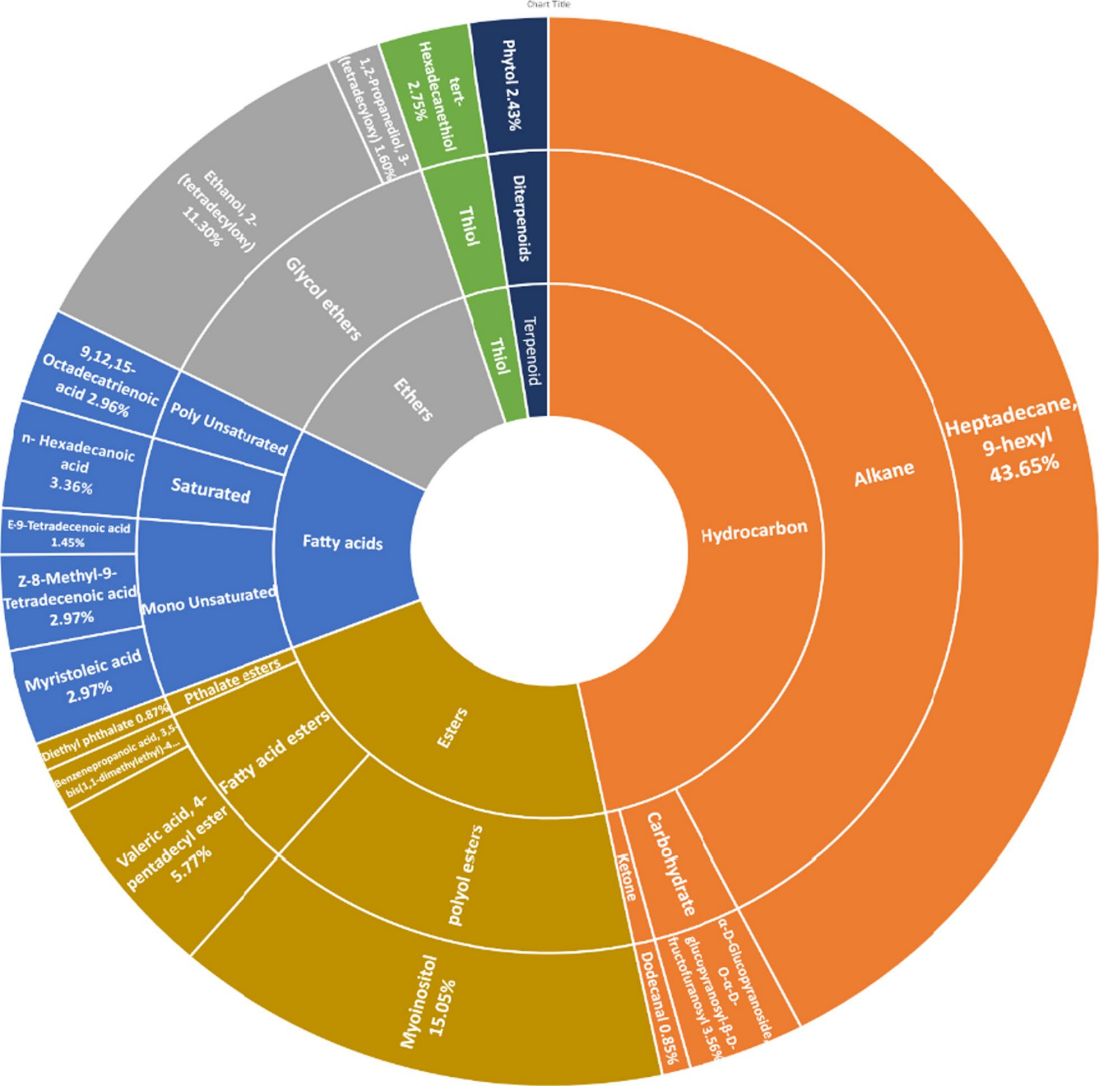
Sunburst chart representing variation in phytoconstituents in *Solanum khasianum* leaf extract

**Fig. 4:**
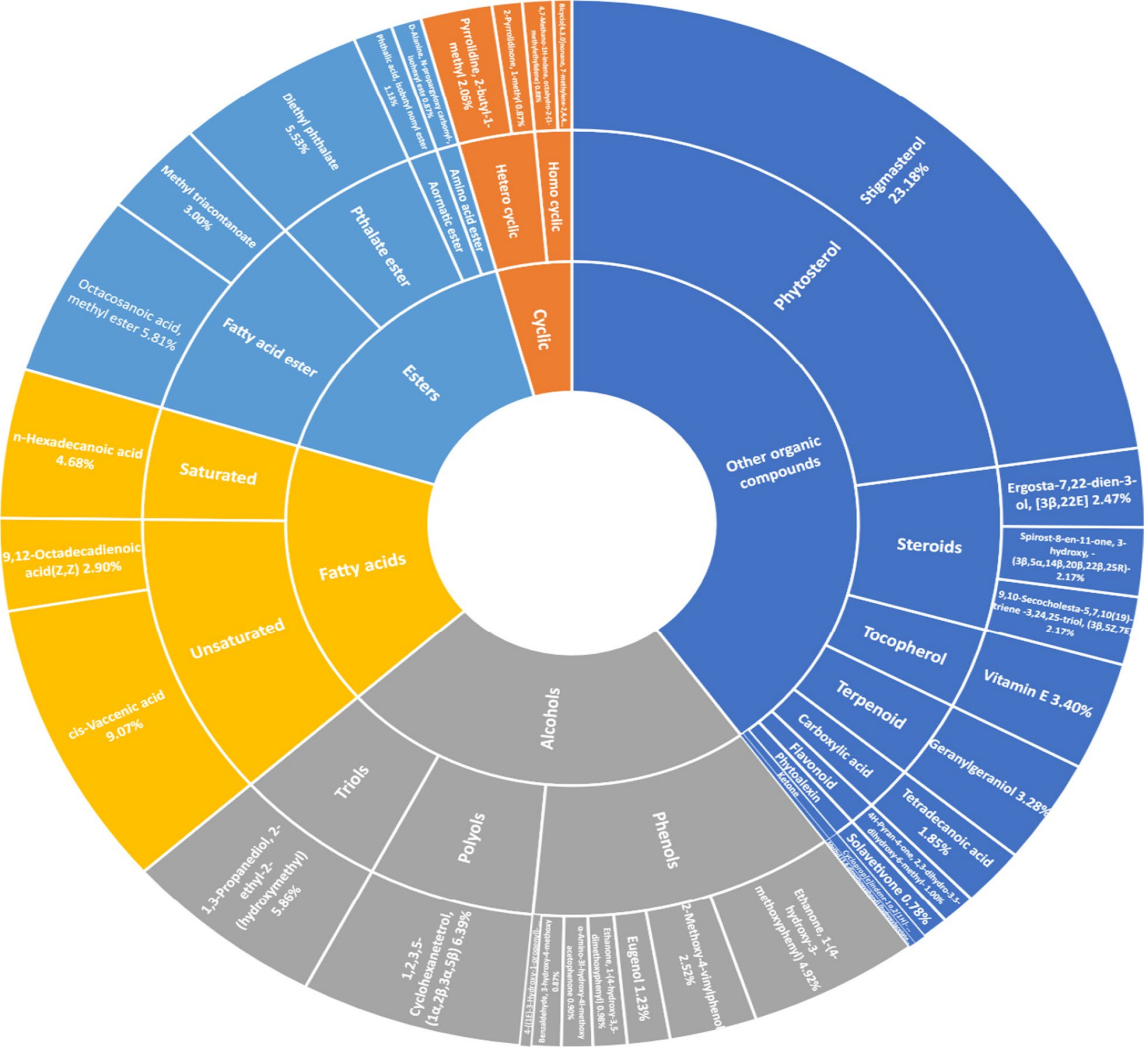
Sunburst chart representing variation in phytoconstituents in *Solanum khasianum* root extract

GC–MS profiling detected potential phytochemicals in *S. khasianum* leaf and root methanolic extracts by their molecular formula and retention time. Sixteen phytoconstituents were detected from leaf extract and thirty-two compounds from root extract by GC–MS (Tables 1 and 2). The compounds identified with high concentration in leaf extract include Heptadecane 9-hexyl (43.65%) and Myoinositol hexaacetate (15.05%), whereas the highest compounds identified in root extract include Stigmasterol (23.18%) and *cis*-Vaccenic acid (9.07%) and presented in Figs. 1 and 2. The diversification of these phytoconstituents was recorded using sunburst graph (Figs. 3 and 4).

**Table 1:** Phytochemical constituents identified in leaf methanolic extracts of *Solanum khasianum* by GC–MS analysis and mass spectra of NIST database

RT	Name of compound	Kovats relative index	Molecular formula	Mwt	Area (%)	Recorded pharmacological activity
13.63	Dodecanal	1387	C_12_H_24_O	184	0.85	Antibacterial, in *pharmaceuticals*
17.47	Diethyl phthalate	1603	C_12_H_14_O_4_	222	0.87	Antimicrobial, antioxidant, plasticizer, estrogenic
20.71	E-9-Tetradecenoic acid	2537	C_14_H_26_O_2_	226	1.45	Analgesic, anti-inflammatory, antioxidant
21.45	Z-8-Methyl-9-Tetradecenoic acid	1676	C_15_H_28_O_2_	240	2.97	No activity reported
21.4	Myristoleic acid	1783	C_14_H_26_O_2_	226	2.97	Cancer preventive
24.03	*n*-Hexadecanoic acid	1972	C_16_H_32_O_2_	256	3.36	Anti-inflammatory, antioxidant, anti-androgenic, hypocholesterolemic, hemolytic nematicide, pesticide, 5-α reductase inhibitor, potent mosquito larvicide, treat rheumatic symptoms
26.20	Phytol	2105	C_20_H_40_O	296	2.43	Antinociceptive, antioxidant, anti-inflammatory, antiallergic, hypolipidemic, anticancer, antimicrobial, cytotoxic, anti-teratogenic, antidiabetic, antispasmodic, anticonvulsant, disinfectant, antidiuretic
26.70	9,12,15-Octadecatrienoic acid	2125	C_18_H_30_O_2_	278	2.96	Analgesic, anesthetic, anticonvulsant, anti-inflammatory, antioxidant, anti-pyretic, antibacterial, cancer preventive, hypocholesterolemic, hepatoprotective, nematicide, antihistaminic and reduce complications in Covid-19 patients
28.68	α-D-Glucopyranoside, O-α-D-glucopyranosyl-β-D-fructofuranosyl	1926	C_18_H_32_O_16_	504	3.56	Cardioprotective, neuroprotective, antidiabetic, antiosteoporotic, anti-inflammatory, antistress
29.89	1,2-Propanediol, 3-(tetradecyloxy)	1603	C_17_H_36_O_3_	288	1.60	Antifungal activity
30.34	tert-Hexadecanethiol	1522	C_16_H_34_S	258	2.76	Antitumor, antioxidant, antifungal, insecticidal
30.70	Ethanol, 2-(tetradecyloxy)	1930	C_16_H_34_O_2_	258	11.3	No activity reported
31.56	Heptadecane, 9-hexyl	2243	C_23_H_48_	324	43.65	Antifungal agent
32.40	Myoinositol, Hexaacetate	2084	C_18_H_42_O_12_	432	15.05	Precursor of several metabolic pathways, co-factors of enzymes, messenger molecule in signal transduction, reduce liver and myocardial lipid content, alternative of metformin
32.77	Valeric acid, 4-pentadecyl ester	2112	C_20_H_40_O_2_	312	5.77	No activity reported
34.80	Benzenepropanoic acid, 3,5-bis(1,1-dimethylethyl)-4-hydroxy-,octadecyl ester	1943	C_35_H_62_O_3_	530	1.37	Antifungal and antioxidant

**Table 2:** Phytochemical constituents identified in root methanolic extracts of *Solanum khasianum* by GC–MS analysis and mass spectra of NIST database

RT	Compound	Kovats relative index	Molecular formula	Mwt	Area	Biological activity
4.84	2-Pyrrolidinone, 1-methyl	1646	C_5_H_9_NO	99	0.87	Anticancer, antioxidant, antibacterial, antifungal, anticonvulsant, surfactant
5.34	d-Alanine, *N*-propargyloxycarbonyl-, isohexyl ester	1725	C_13_H_21_NO_4_	255	0.87	No activity reported
5.46	Pyrrolidine, 2-butyl-1-methyl	1072	C_9_H_19_N	141	2.06	No activity reported
6.41	4*H*-Pyran-4-one, 2,3-dihydro-3,5-dihydroxy-6-methyl-	1134	C_6_H_8_O_4_	144	1.0	Anti-diabetic, antioxidant, antibacterial, melanin production inhibitor
9.54	2-Methoxy-4-vinylphenol	1315	C_9_H_10_O_2_	150	2.52	Antioxidant, antimicrobial, anti-inflammatory
10.35	Eugenol	1356	C_10_H_12_O_2_	164	1.23	Antioxidant, antimicrobial, anti-proliferative, anti-inflammatory
11.20	Methyl (3,4-dimethoxyphenyl)(hydroxy)acetate	1700	C_11_H_14_O_5_	226	0.19	No activity reported
11.33	Benzaldehyde, 3-hydroxy-4-methoxy	1401	C_8_H_8_O_3_	152	0.87	Antimicrobial activity
12.50	1,3-Propanediol, 2-ethyl-2-(hydroxymethyl)-	1261	C_6_H_14_O_3_	134	5.86	Antioxidant, Antimicrobial
13.05	Ethanone, 1-(4-hydroxy-3-methoxyphenyl)	1447	C_9_H_10_O_3_	166	4.92	Anti-inflammatory, antioxidant, non-steroidal, enzyme inhibitor, food additive
14.95	Diethyl phthalate	1603	C_12_H_14_O_4_	222	5.53	Antimicrobial, antioxidant, plasticizer, estrogenic
16.25	1,2,3,5-Cyclohexanetetrol, (1α,2β,3α,5β)	1472	C_6_H_12_O_4_	148	6.39	No activity reported
16.77	α-Amino-3-hydroxy-4-methoxyacetophenone	2819	C_9_H_11_NO_3_	181	0.9	No activity reported
17.67	Ethanone, 1-(4-hydroxy-3,5-dimethoxyphenyl)	1741	C_10_H_12_O_4_	196	0.98	Anti-inflammatory, antioxidant, non-steroidal, enzyme inhibitors, food additive
17.87	4-((1E)-3-Hydroxy-1-propenyl)-2-methoxyphenol	1688	C_10_H_12_O_3_	180	0.31	Antimicrobial, antioxidant, anti-inflammatory, analgesic
17.98	Tetradecanoic acid	1761	C_14_H_28_O_2_	228	1.85	Cancer preventive, antioxidant, nematicide, lubricant, hypocholesterolemic
18.95	Solavetivone	1779	C_15_H_22_O	218	0.78	Antibacterial, fungitoxic, antimicrobial, weak cytotoxic activity
19.47	Cycloprop[e]indene-1a,2[1*H*]-dicarboxaldehyde, 3a,4,5,6,6a,6b-hexahydro-5,5,6b-trimethyl-, [1aα,3aβ,6aβ,6bα]-[+]	1734	C_15_H_20_O_2_	232	0.33	No activity reported
19.81	Phthalic acid, isobutyl nonyl ester	2470	C_21_H_32_O_4_	348	1.13	Efficient in curing chronic cardiovascular, cerebrovascular diseases, anti-tumor, anti-inflammatory, antibacterial
20.35	4,7-Methano-1*H*-indene, octahydro-2-(1-methylethylidene)	1078	C_13_H_20_	176	0.88	No activity reported
20.69	Bicyclo[4.3.0]nonane, 7-methylene-2,4,4-trimethyl-2-vinyl	1085	C_15_H_24_	204	0.54	No activity reported
21.36	*n*-Hexadecanoic acid	1975	C_16_H_32_O_2_	256	4.68	Anti-inflammatory, antioxidant, antiandrogenic, hypocholesterolemic, nematicide, pesticide, hemolytic, 5-α reductase inhibitor, mosquito larvicide
23.45	Ergosta-7,22-dien-3-ol, [3β,22E]	3202	C_28_H_46_O	398	2.47	No activity reported
23.91	9,12-Octadecadienoic acid(Z,Z)	2134	C_18_H_32_O_2_	280	2.9	Anticarcinogenic, antioxidant, anti-inflammatory, antiatherogenic
24.02	*cis*-Vaccenic acid	2162	C_18_H_34_O_2_	282	9.07	Anticarcinogenic effect
25.52	Geranylgeraniol	2201	C_20_H_34_O	290	3.28	Anti-tumorigenic, anti-inflammatory, neuroprotective
28.39	Stigmasterol	3170	C_29_H_48_O	412	23.18	Anti-inflammatory, antioxidant, antimicrobial, sedative activity
29.05	Methyl triacontanoate	3317	C_31_H_62_O_2_	466	3.0	No activity reported
29.25	Vitamin E	3111	C_29_H_50_O_2_	430	3.4	Anticancer, antidiabetic, antioxidant, anti-inflammatory, antiaging, analgesic, antidermatitic, antileukemia, antibronchitic, anticoronary, vasodilator, hepatoprotective, hypocholesterolemic, antiulcerogenic, antispasmodic
29.44	Octacosanoic acid, methyl ester	3112	C_29_H_58_O_2_	438	5.81	No activity reported
30.66	9,10-Secocholesta-5,7,10(19)-triene-3,24,25-triol, (3β,5Z,7E)	2642	C_27_H_44_O_3_	416	2.17	Biocide, anti-corrosion agents
30.66	Spirost-8-en-11-one, 3-hydroxy, -(3β,5α,14β,20β,22β,25R)-	3044	C_27_H_40_O_4_	428	2.17	Anticancer, Estrogenic, progesterogenic, anti-inflammatory

Table 3 shows the antibacterial activity of *S. khasianum* leaf and root extracts. The root methanol extract showed the highest inhibition zone at 80 µg/ml of 16 ± 0.15 mm for *B. sphaericus,* 21 ± 0.18 mm for *Escherichia coli*, 17 ± 0.02 mm for *Staphylococcus aureus* and 19 ± 0.18 mm for *Pseudomonas aeruginosa.* Leaf extract at 80 µg/ml concentration showed 15 ± 0.14 mm for *B. sphaericus,* 16 ± 0.16 mm for *Escherichia coli*, 15 ± 0.01 mm for *Staphylococcus aureus* and 17 ± 0.11 mm for *Pseudomonas aeruginosa.*

**Table 3:** Antibacterial activity of *Solanum khasianum* extracts on tested bacteria

Test organism	Leaf extract concentration (µg/ml)					Root extract concentration (µg/ml)				
	80	60	40	20	Std	80	60	40	20	Std
*B. sphaericus*	15 ± 0.14	13 ± 0.19	12 ± 0.07	10 ± 0.14	24 ± 0.12	16 ± 0.15	15 ± 0.14	13 ± 0.16	12 ± 0.12	28 ± 0.11
*E. coli*	16 ± 0.16	15 ± 0.22	13 ± 0.13	12 ± 0.19	27 ± 0.15	21 ± 0.18	18 ± 0.20	16 ± 0.24	13 ± 0.16	24 ± 0.09
*S. aureus*	15 ± 0.01	13 ± 0.14	12 ± 0.17	11 ± 0.02	25 ± 0.08	17 ± 0.02	14 ± 0.12	13 ± 0.11	11 ± 0.15	24 ± 0.16
*P. aeruginosa*	17 ± 0.11	15 ± 0.12	14 ± 0.23	13 ± 0.15	20 ± 0.19	19 ± 0.18	16 ± 0.19	15 ± 0.21	13 ± 0.22	27 ± 0.13

## Discussion

Accurate certification and studies of phytoconstituents are increasing periodically, as they are repositories of several potent drugs. Gas chromatography and mass spectroscopy (GC–MS) has been validated to be a significant tool for bioprospecting of plant bioactive compounds. However, diethyl phthalate and *n*-hexadecanoic acid were identified to be common in leaf and root extract of *S. khasianum.* Other organic compounds in leaf extract that are accountable for their wide use in medicinal aid include: Dodecanal, reported to possess highest antibacterial activity (Faridha Begum et al. 2016). Benzenepropanoic acid, 3,5-bis(1,1-dimethylethyl)-4-hydroxy-,octadecyl ester shows strong antifungal and antioxidant activities in *Azadirachta* and *Thesium humile* (Akpuaka et al. 2013; Belakhdar et al. 2015).

The remaining bioactive compounds analyzed were as follows: Diethyl phthalate, a phytoconstituent well known for its antimicrobial, antioxidant, plasticizer and estrogenic activities in *Ceropegia bulbosa* Roxb (Arora and Meena 2017). E-9-Tetradecenoic acid is reported to have analgesic, anti-inflammatory and antioxidant properties in *Cassia angustifolia* (Al-Marzoqi et al. 2016). The bioactive compound, Myristoleic acid reported in Sesame Seeds was known to prevent cancer (Bhatnagar and Gopala Krishna 2009).

The bioactive molecule *n*-Hexadecanoic acid has reported to have multiple biological properties in *Vitex negundo* (Kumar et al. 2019; Enerijiofi et al. 2021). The phytol, a bioactive compound reported earlier in several species like *Hydrilla verticillate, Gracilaria edulis* and *Carissa carandas* with diversified medicinal uses (Prabha et al. 2019; Rao et al. 2019). The compound 9,12,15-Octadecatrienoic acid was known to possess several biological properties like analgesic, anesthetic, anticonvulsant, anti-inflammatory, antioxidant, anti-pyretic, antibacterial (Kalaivani et al. 2012); anticancer, antihistaminic, hepatoprotective, hypocholesterolemic, nematicide (Rao et al. 2019) in *Andrographis paniculata* and *Carissa carandas* and also known to reduce complications in Covid-19 patients (Weill et al. 2020). α-d-Glucopyranoside, *O*-α-d-glucopyranosyl-β-d-fructofuranosyl, a phytochemical compound also found in *Cyperus alternifolius* have cardioprotective, neuroprotective, antidiabetic, antiosteoporotic, anti-inflammatory and antistress properties (Al-Gara et al. 2019)*.* The 1,2-Propanediol, 3-(tetradecyloxy), a phytoconstituent reported to have antifungal activity (Sundberg and Faergemann, 2008), whereas the compound tert-Hexadecanethiol was known for its antitumor activity in *Malaxis acuminta* (Raval et al. 2016); antioxidant, antifungal and insecticidal activities in *Capsicum annuum* (Sathya et al. 2016). Another bioactive molecule Heptadecane, 9-hexyl (Fig. 2), the major bioactive compound of *S. khasianum* leaf extract, known to possess strong antifungal activity in *Senecio coluhuapiensis* (Arancibia et al. 2016). The compound Myoinositol, hexaacetate acts as a precursor of several metabolic pathways, co-factors for enzymes and as messenger molecule in signal transduction (Chhetri, 2019; Kim et al. 2008). The biological activity of some compounds has not yet identified (Table 1).

The chemical profiling of root methanolic extracts of *S. khasianum* identified different bioactive compounds. Among them, more predominant compound identified was stigmasterol, known to possess anti-inflammatory, antioxidant, antimicrobial and sedative activities (Al-Rubaye et al. 2017). The initial compound eluted was 2-Pyrrolidinone, 1-methyl with anticancer, antioxidant, antibacterial, antifungal, anticonvulsant and surfactant properties (Hosseinzadeh et al. 2017). The other bioactive compounds identified were as follows: 4*H*-Pyran-4-one, 2,3-dihydro-3,5-dihydroxy-6-methyl, a ketone reported earlier in *Malva sylvestris*, known to possess several biological properties (Al-Rubaye et al. 2017; Ashwathanarayana and Naika, 2017). 2-Methoxy-4-vinylphenol, a phytoconstituent with antioxidant, antimicrobial, anti-inflammatory properties in *Cassia angustifolia* (Alghamdi et al. 2018). The compound, Eugenol, has several biological properties like antioxidant, antimicrobial (Hamed et al. 2012), anti-proliferative and anti-inflammatory activities (Fujisawa and Murakami 2016).

The bioactive compound Benzaldehyde, 3-hydroxy-4-methoxy, which is known for its antimicrobial activity and inhibits enzymes like 17-β-hydroxysteroid dehydrogenase, testosterone hydroxylase and arylamine-*N*-acetyltransferase (Prabhu et al. 2020). 1,3-Propanediol, 2-ethyl-2-(hydroxymethyl), is one such bioactive molecule with antioxidant and antimicrobial activity in *Erythrina variegata* (Umarani and Nethaji 2021). Ethanone, 1-(4-hydroxy-3-methoxyphenyl) and Ethanone, 1-(4-hydroxy-3,5-dimethoxyphenyl) were the two identified non-steroidal bioactive compounds reported to have anti-inflammatory, antioxidant, enzyme inhibitor properties and also employed as food additive (Ashwathanarayana and Naika 2017). The compound 4-((1E)-3-Hydroxy-1-propenyl)-2-methoxyphenol, has been reported to have diverse biological activities like antimicrobial, antioxidant, anti-inflammatory and analgesic (Mostafa et al. 2020). Tetradecanoic acid was identified as a cancer preventive, antioxidant, nematicide, lubricant and hypocholesterolemic in *Ceropegia bulbosa* (Arora and Meena 2017). Solavetivone, a phytoconstituent of tobacco and *Solanum erianthum*, has fungitoxic, antimicrobial and weak cytotoxic activities (Chen et al. 2013). Similarly, a compound phthalic acid, isobutyl nonyl ester was observed to be efficient in curing persistent cardiac and cerebrovascular problems, cancer, inflammation and bacterial infections (Ma et al. 2015). The compound 9,12-Octadecadienoic acid (Z,Z) was known to possess anticarcinogenic, antioxidant, anti-inflammatory and antiatherogenic properties (Arora and Meena 2017).

The second highest compound, *cis*-vaccenic acid, was well known for its anti-carcinogenic effect in *Origanum vulgare* (Al-Tameme et al. 2015). Similarly, geranylgeraniol (Ho et al. 2018) and vitamin E (Arora et al. 2017) were reported to have several biological properties. The compound 9,10-Secocholesta-5,7,10(19)-triene-3,24,25-triol, (3β,5Z,7E), acts as biocide and anti-corrosion agent in *Piper nigrum* (Mohammed et al. 2016). Spirost-8-en-11-one, 3-hydroxy, -(3β,5α,14β,20β,22β,25R) was found to possess anticancer (Rajendran et al. 2017), estrogenic, progesterogenic and anti-inflammatory effects (Gopu et al. 2021). Among the bioactive compounds identified in the root methanolic extracts of *S. khasianum,* the biological activity of some compounds was not yet identified and reported (Table 2).

The *S. khasianum* leaf methanolic extracts showed high antibacterial activity against *P. aeruginosa* in concentration-dependent manner, followed by *E. coli, B. sphaericus* and *S. aureus* (Fig. 3), whereas the root methanolic extract exhibited high antibacterial activity against *E. coli,* followed by *P. aeruginosa, S. aureus* and *B. sphaericus.* The result indicates that the *S. khasianum* root extract exhibited remarkable antibacterial property against *P. aeruginosa* and *E. coli.* Therefore, root methanolic extract of *S. khasianum* was considered as the most effective extract than leaf extract with regard to high anti-bacterial activity (Pavani and Shasthree 2021). This indicates that the root extract had more antibacterial compounds than leaf extract. Our results were in accordance with the reports on *Momordica cymbalaria* (Chaitanya and Pavani 2021). This study confirms that the *S. khasianum* extracts have significant antibacterial activity against tested bacteria.

## Conclusions

The GC–MS analysis revealed the presence of 16 bioactive compounds in leaf methanolic extract and 32 bioactive compounds in root methanolic extract of *S. khasianum* based on their retention time, molecular weight, peak area and MS fragment ions generated. Heptadecane, 9-hexyl and stigmasterol were the predominant potential bioactive compounds identified in leaf and root extract. These extracts have shown high antibacterial activity against gram-positive and gram-negative bacteria. This study confirmed the presence of various biomolecules with significant biological properties, thereby confirming the medicinal claim and use of *Solanum khasianum* and making it a potential source of medicines.

## Data Availability

The datasets generated and/or analyzed during the current study are available from the corresponding author on reasonable request.

## Abbreviations

GC–MS: Gas chromatography and mass spectroscopy
W/V: Weight/volume
rpm: Rotation per minute
µl: Microliter
ml: Milliliter
LB: Luria Bertani
µg/ml: Microgram per milliliter
DMSO: Dimethyl sulfoxide
Hrs: Hours
SD: Standard deviation
mm: Millimeter
